# Supplementary material for: Elemental Analysis and Phenolic Profiles of Selected Italian Wines
Source: Foods. 2021 Jan 13;10(1):158. doi: 10.3390/foods10010158 (PMC7828534; doi:10.3390/foods10010158)
Supplement: Supplementary file 1 [file foods-10-00158-s001.pdf]

Supplementary Materials

Table S1. Metals with stdev

	**W1**	**W2**	**W3**	**W4**	**W5**	**W6**	**W7**	**W8**	**W9**	**W10**	**W11**	**W12**	**W13**
**As**	<LOD	<LOD	19.84±0.03	<LOD	<LOD	<LOD	<LOD	<LOD	<LOD	<LOD	<LOD	<LOD	<LOD
**Ba**	32.6±0.3	33.8±0.4	26.1±0.2	123.3±0.9	79.8±0.8	79.1±0.7	83.3±0.6	239.7±0.8	58.1±0.4	77.7±0.3	32.1±0.3	31.7±0.5	122.1±0.8
**Cd**	0.365±0.004	1.553±0.002	0.939±0.002	1.254±0.003	0.386±0.005	1.367±0.008	0.733±0.004	0.050±0.002	0.976±0.002	1.644±0.006	<LOD	0.593±0.004	0.375±0.003
**Co**	3.64±0.04	2.09±0.02	0.98±0.03	4.40±0.06	2.77±0.02	1.83±0.02	2.42±0.02	2.68±0.03	7.34±0.09	7.78±0.07	2.12±0.04	1.61±0.02	2.60±0.03
**Cr**	<LOD	<LOD	<LOD	<LOD	4.322±0.005	3.030±0004	<LOD	6.417±0.006	15.41±0.02	14.82±0.05	11.12±0.04	9.15±0.02	3.111±0.004
**Pb**	<LOD	56.0±0.8	<LOD	20.8±0.9	80±2	11.7±0.6	2.20±0.05	175±3	45.0±0.8	<LOD	0.830±0.007	0.538±0.006	14.9±0.3
**Sb**	32.8±0.5	8.59±0.09	19.1±0.2	18.±0.2	67.5±0.5	37.9±0.2	9.58±0.05	46.5±0.3	44.8±0.4	18.50±0.08	7.26±0.03	44.3±0.2	29.6±0.4
**Se**	<LOD	<LOD	<LOD	<LOD	1.45±0.09	0.30±0.05	0.23±0.08	25.7±0.4	2.87±0.2	2.06±0.04	2.91±0.8	2.38±0.07	2.99±0.06
**Ni**	4.17±0.05	46.0±0.2	0.569±0.005	30.9±0.3	15.3±0.2	5.77±0.02	35.2±0.3	13.86±0.04	50.6±0.7	17.9±0.2	9.99±0.03	0.197±0.007	5.00±0.02
**Al**	566±5	260.1±0.5	155.3±0.4	720±5	447±4	290.7±2	26.5±0.4	155.4±0.9	444±4	329±3	1027±10	567.7±8	236±3
**Cu**	97.5±0.8	469±3	36.3±0.9	10.07±0.05	<LOD	<LOD	<LOD	<LOD	21.19±0.07	7.73±0.03	0.813±0.05	49.3±0.8	50.2±1.0
**Mn**	735±30	613±10	642±6	1016±6	762±3	743±5	839±8	1634±30	1732±20	1472±20	887±10	771±6	1299±10
**V**	4.18±0.02	1.894±0.007	0.574±0.002	<LOD	1.530±0.005	<LOD	1.948±0.006	3.179±0.004	3.235±0.007	2.304±0.003	0.810±0.002	0.729±0.002	<LOD
**Zn**	468.4±0.7	1359±8	423.8±5	622.2±3	1356±10	607±7	298±3	969±8	1278±9	916±4	519±2	537±3	897±4
**Ca**[a]	70.7±0.9	76.9±1.0	71.7±0.8	111.2±1.0	62.5±0.7	83.9±0.4	50.7±0.4	67.5±0.7	62.8±0.5	98.1±0.6	76.4±0.2	99.9±0.3	71.1±0.2
**Fe**[a]	0.134±0.002	0.077±0.05	0.050±0.002	0.786±0.004	0.525±0.006	0.520±0.007	0.593±0.002	1.082±0.008	1.809±0.007	0.688±0.004	0.371±0.002	0.198±0.002	0.870±0.003
**K**[a]	489±5	707±5	663±7	1152±10	609±3	719±4	965±8	1119±9	567±6	867±8	700±7	1174±10	1111±9
**Mg**[a]	55.3±0.8	52.9±0.4	52.4±0.5	77.4±0.2	70.3±0.8	71.0±0.4	82.1±0.5	102.0±0.8	64.0±0.3	68.4±0.4	69.11±0.09	60.4±0.2	94.3±0.3
**Na**[a]	7.75±0.02	14.13±0.06	9.32±0.04	38.24±0.02	29.61±0.08	27.41±0.05	11.69±0.02	16.88±0.04	21.60±0.02	26.49±0.06	9.36±0.04	17.62±0.02	22.08±0.04
**Rb**[a]	2.69±0.04	2.96±0.05	3.084±0.006	3.83±0.05	3.90±0.08	3.85±0.04	5.07±0.02	7.38±0.07	4.33±0.08	3.96±0.08	4.08±0.04	3.59±0.02	5.63±0.07

Table S2. Correlation coefficients among elements in analyzed wine samples.

	As	Ba	Cd	Co	Cr	Pb	Sb	Se	Ni	Al	Cu	Mn	V	Zn	Ca	Fe	K	Mg	Na	Rb
**As**	1.0000	-.2672	.0830	-.3265	-.2671	-.1865	-.1738	-.1333	-.3076	-.2739	-.0492	-.2854	-.2348	-.2982	-.0968	-.3361	-.2115	-.3626	-.3284	-.2643
		p=.378	p=.787	p=.276	p=.378	p=.542	p=.570	p=.664	p=.307	p=.365	p=.873	p=.345	p=.440	p=.322	p=.753	p=.262	p=.488	p=.223	p=.273	p=.383
**Ba**	-.2672	1.0000	-.2544	.0797	-.0297	.7466	.2792	.8166	.0213	-.3163	-.3050	.5964	.1017	.1825	-.0500	.5128	.5745	.8954	.3583	.8795
	p=.378		p=.402	p=.796	p=.923	p=.003	p=.356	p=.001	p=.945	p=.292	p=.311	p=.031	p=.741	p=.551	p=.871	p=.073	p=.040	p=.001	p=.229	p=.001
**Cd**	.0830	-.2544	1.0000	.3644	-.0159	-.3036	-.3154	-.4625	.4230	-.2538	.3659	-.0744	-.1585	.1726	.4411	-.0347	-.0787	-.3990	.4030	-.4474
	p=.787	p=.402		p=.221	p=.959	p=.313	p=.294	p=.112	p=.150	p=.403	p=.219	p=.809	p=.605	p=.573	p=.131	p=.910	p=.798	p=.177	p=.172	p=.125
**Co**	-.3265	.0797	.3644	1.0000	.6376	-.0204	.0562	-.0252	.4700	.1113	-.1797	.7024	.4590	.3620	.1808	.6433	-.1255	.0046	.3907	.0229
	p=.276	p=.796	p=.221		p=.019	p=.947	p=.855	p=.935	p=.105	p=.717	p=.557	p=.007	p=.115	p=.224	p=.554	p=.018	p=.683	p=.988	p=.187	p=.941
**Cr**	-.2671	-.0297	-.0159	.6376	1.0000	.0374	.1913	.1958	.1161	.3133	-.3136	.6492	.2208	.2940	.1406	.5254	-.0415	.0105	.1072	.1695
	p=.378	p=.923	p=.959	p=.019		p=.904	p=.531	p=.521	p=.706	p=.297	p=.297	p=.016	p=.469	p=.330	p=.647	p=.065	p=.893	p=.973	p=.727	p=.580
**Pb**	-.1865	.7466	-.3036	-.0204	.0374	1.0000	.4857	.8481	.1940	-.2641	.0649	.4273	.3643	.5676	-.2816	.3874	.1524	.5079	.1217	.6468
	p=.542	p=.003	p=.313	p=.947	p=.904		p=.092	p=.001	p=.525	p=.383	p=.833	p=.145	p=.221	p=.043	p=.351	p=.191	p=.619	p=.076	p=.692	p=.017
**Sb**	-.1738	.2792	-.3154	.0562	.1913	.4857	1.0000	.3153	-.2126	-.0573	-.3290	.2211	.1942	.4245	-.1541	.3296	-.0744	.1633	.3437	.2109
	p=.570	p=.356	p=.294	p=.855	p=.531	p=.092		p=.294	p=.486	p=.852	p=.272	p=.468	p=.525	p=.148	p=.615	p=.271	p=.809	p=.594	p=.250	p=.489
**Se**	-.1333	.8166	-.4625	-.0252	.1958	.8481	.3153	1.0000	-.0882	-.2103	-.1843	.5720	.3423	.1939	-.1681	.3784	.3738	.6529	-.0723	.8137
	p=.664	p=.001	p=.112	p=.935	p=.521	p=.001	p=.294		p=.774	p=.490	p=.547	p=.041	p=.252	p=.526	p=.583	p=.202	p=.208	p=.016	p=.814	p=.001
**Ni**	-.3076	.0213	.4230	.4700	.1161	.1940	-.2126	-.0882	1.0000	-.1157	.3960	.2825	.3033	.4937	-.1794	.4791	-.1174	-.0467	.1765	.0203
	p=.307	p=.945	p=.150	p=.105	p=.706	p=.525	p=.486	p=.774		p=.707	p=.180	p=.350	p=.314	p=.086	p=.558	p=.098	p=.703	p=.880	p=.564	p=.948
**Al**	-.2739	-.3163	-.2538	.1113	.3133	-.2641	-.0573	-.2103	-.1157	1.0000	-.1349	-.1069	-.1319	-.1078	.4384	-.0806	-.1249	-.2469	.0988	-.3418
	p=.365	p=.292	p=.403	p=.717	p=.297	p=.383	p=.852	p=.490	p=.707		p=.660	p=.728	p=.668	p=.726	p=.134	p=.794	p=.684	p=.416	p=.748	p=.253
**Cu**	-.0492	-.3050	.3659	-.1797	-.3136	.0649	-.3290	-.1843	.3960	-.1349	1.0000	-.3467	.1261	.4026	.0020	-.3814	-.1915	-.4310	-.2587	-.3750
	p=.873	p=.311	p=.219	p=.557	p=.297	p=.833	p=.272	p=.547	p=.180	p=.660		p=.246	p=.681	p=.173	p=.995	p=.198	p=.531	p=.141	p=.393	p=.207
**Mn**	-.2854	.5964	-.0744	.7024	.6492	.4273	.2211	.5720	.2825	-.1069	-.3467	1.0000	.3670	.3715	-.0428	.8820	.2561	.5551	.2672	.6645
	p=.345	p=.031	p=.809	p=.007	p=.016	p=.145	p=.468	p=.041	p=.350	p=.728	p=.246		p=.217	p=.211	p=.890	p=.001	p=.398	p=.049	p=.377	p=.013
**V**	-.2348	.1017	-.1585	.4590	.2208	.3643	.1942	.3423	.3033	-.1319	.1261	.3670	1.0000	.2487	-.4145	.2584	-.3947	-.0776	-.3595	.0956
	p=.440	p=.741	p=.605	p=.115	p=.469	p=.221	p=.525	p=.252	p=.314	p=.668	p=.681	p=.217		p=.413	p=.159	p=.394	p=.182	p=.801	p=.228	p=.756
**Zn**	-.2982	.1825	.1726	.3620	.2940	.5676	.4245	.1939	.4937	-.1078	.4026	.3715	.2487	1.0000	-.1417	.4135	-.2029	.0319	.3532	.1202
	p=.322	p=.551	p=.573	p=.224	p=.330	p=.043	p=.148	p=.526	p=.086	p=.726	p=.173	p=.211	p=.413		p=.644	p=.160	p=.506	p=.918	p=.237	p=.696
**Ca**	-.0968	-.0500	.4411	.1808	.1406	-.2816	-.1541	-.1681	-.1794	.4384	.0020	-.0428	-.4145	-.1417	1.0000	-.1749	.4219	-.1592	.5161	-.3029
	p=.753	p=.871	p=.131	p=.554	p=.647	p=.351	p=.615	p=.583	p=.558	p=.134	p=.995	p=.890	p=.159	p=.644		p=.568	p=.151	p=.603	p=.071	p=.314
**Fe**	-.3361	.5128	-.0347	.6433	.5254	.3874	.3296	.3784	.4791	-.0806	-.3814	.8820	.2584	.4135	-.1749	1.0000	.1364	.5131	.4075	.5841
	p=.262	p=.073	p=.910	p=.018	p=.065	p=.191	p=.271	p=.202	p=.098	p=.794	p=.198	p=.001	p=.394	p=.160	p=.568		p=.657	p=.073	p=.167	p=.036
**K**	-.2115	.5745	-.0787	-.1255	-.0415	.1524	-.0744	.3738	-.1174	-.1249	-.1915	.2561	-.3947	-.2029	.4219	.1364	1.0000	.6380	.3188	.5592
	p=.488	p=.040	p=.798	p=.683	p=.893	p=.619	p=.809	p=.208	p=.703	p=.684	p=.531	p=.398	p=.182	p=.506	p=.151	p=.657		p=.019	p=.288	p=.047
**Mg**	-.3626	.8954	-.3990	.0046	.0105	.5079	.1633	.6529	-.0467	-.2469	-.4310	.5551	-.0776	.0319	-.1592	.5131	.6380	1.0000	.2872	.9343
	p=.223	p=.001	p=.177	p=.988	p=.973	p=.076	p=.594	p=.016	p=.880	p=.416	p=.141	p=.049	p=.801	p=.918	p=.603	p=.073	p=.019		p=.341	p=.001
**Na**	-.3284	.3583	.4030	.3907	.1072	.1217	.3437	-.0723	.1765	.0988	-.2587	.2672	-.3595	.3532	.5161	.4075	.3188	.2872	1.0000	.0907
	p=.273	p=.229	p=.172	p=.187	p=.727	p=.692	p=.250	p=.814	p=.564	p=.748	p=.393	p=.377	p=.228	p=.237	p=.071	p=.167	p=.288	p=.341		p=.768
**Rb**	-.2643	.8795	-.4474	.0229	.1695	.6468	.2109	.8137	.0203	-.3418	-.3750	.6645	.0956	.1202	-.3029	.5841	.5592	.9343	.0907	1.0000
	p=.383	p=.001	p=.125	p=.941	p=.580	p=.017	p=.489	p=.001	p=.948	p=.253	p=.207	p=.013	p=.756	p=.696	p=.314	p=.036	p=.047	p=.001	p=.768	

Table S3. Polyphenols, TPC, and RSA stdev.

Non-anthocyanins (mg/L)	**W1**	**W2**	**W3**	**W4**	**W5**	**W6**	**W7**	**W8**	**W9**	**W10**	**W11**	**W12**	**W13**
Hydroxybenzoic acids													
**Gallic acid**	1.99±0.30	1.09±0.16	–	41.73±6.26	38.20±5.73	24.22±3.63	74.36±11.15	81.35±12.20	4.40±0.66	2.53±0.38	2.82±0.42	3.24±0.49	74.62±11.19
**Protocatechuic acid**	0.53±0.05	0.57±0.06	0.57±0.06	2.63±0.27	1.33±0.13	1.78±0.18	7.27±0.73	5.41±0.55	1.70±0.17	1.41±0.14	1.57±0.16	1.30±0.13	8.23±0.83
**Gentisic acid**	1.45±0.15	3.24±0.33	3.36±0.34	1.52±0.15	2.90±0.29	1.47±0.15	1.01±0.10	0.87±0.09	1.47±0.15	0.49±0.05	0.71±0.07	1.26±0.13	–
**Ellagic acid**	0.71±0.11	0.51±0.08	0.65±0.10	1.17±0.18	0.81±0.12	1.01±0.15	25.24±3.79	24.41±3.66	1.36±0.20	0.74±0.11	0.49±0.07	0.61±0.09	29.02±4.35
Hydroxicinnamic acids													
**Sinapic acid**	–	–	–	–	–	–	–	–	–	–	–	23.67±2.62	–
**Caffeic acid**	0.59±0.07	0.67±0.08	1.36±0.16	8.79±1.04	9.16±1.09	5.47±0.65	4.58±0.54	7.18±0.85	5.78±0.69	2.58±0.31	1.01±0.12	0.95±0.11	5.44±0.65
**Chlorogenic acid**	0.040±0.004	0.030±0.003	0.040±0.004	0.040±0.004	0.07±0.01	0.10±0.01	–	0.07±0.01	0.030±0.003	0.19±0.02	0.12±0.01	0.10±0.01	0.20±0.02
***p*-Coumaric acid**	1.12±0.07	2.03±0.12	1.46±0.09	2.64±0.16	3.02±0.18	2.99±0.18	10.34±0.62	4.63±0.28	1.80±0.11	1.65±0.10	1.60±0.10	4.30±0.26	5.61±0.34
Flavanols													
**Gallocatechin**	–	–	–	1.34±0.13	3.29±0.33	5.37±0.54	–	–	–	–	–	–	0.93±0.10
**Epigallocatechin gallate**	–	–	0.86±0.09	–	–	–	1.02±0.11	1.63±0.17	–	0.91±0.09	–	1.48±0.15	1.31±0.14
**Catechin**	–	–	–	4.23±0.23	5.95±0.33	10.40±0.58	25.93±1.44	16.98±0.94	3.63±0.20	–	1.49±0.08	1.69±0.09	26.67±1.48
**Gallocatechin gallate**	–	–	–	–	0.52±0.05	–	2.48±0.24	–	–	–	1.10±0.10	0.35±0.03	1.94±0.19
Flavonols													
**Myricetin**	–	–	–	–	–	–	0.39±0.02	0.41±0.02	0.020±0.001	–	–	–	0.28±0.01
**Rutin**	–	–	–	0.020±0.001	–	0.010±0.001	–	–	–	0.030±0.001	–	–	0.020±0.001
**Astragalin**	–	–	–	0.010±0.001	–	–	–	0.010±0.001	–	0.010±0.001	–	–	–
**Hyperoside**	–	–	–	–	–	0.010±0.001	0.20±0.01	0.26±0.01	–	–	–	0.010±0.001	0.47±0.02
**Galangin**	1.21±0.18	0.88±0.13	0.60±0.09	0.46±0.07	0.36±0.05	0.20±0.03	0.24±0.04	0.19±0.03	0.18±0.03	–	–	–	–
Flavones													
**Chrysin**	0.60±0.06	–	–	–	–	–	–	–	–	–	–	–	–
**Luteolin**	–	–	–	–	–	–	–	0.10±0.01	–	–	–	–	–
**Cynaroside**	–	–	–	0.67±0.01	–	–	0.48±0.01	0.53±0.01	0.50±0.01	0.57±0.01	–	–	0.35±0.01
**Apigetrin**	–	–	–	0.010±0.001	–	–	0.010±0.001	0.010±0.001	0.010±0.001	0.010±0.001	–	–	0.010±0.001

**Apigenin**	0.060±0.005	–	0.030±0.002	–	–	–	–	–	–	–	–	–	–
Hydroxycoumarins													
**Aesculin**	–	–	0.050±0.002	–	–	–	0.65±0.03	0.69±0.03	–	–	–	0.44±0.02	0.87±0.04
Stilbenes													
**Resveratrol**	–	–	–	–	–	–	4.00±0.17	–	–	–	–	–	–
**Hesperetin**	–	–	0.090±0.005	0.050±0.003	–	–	0.71±0.04	0.44±0.03	–	0.10±0.01	–	–	0.41±0.02
Dihydrochalcones													
**Phlorizin**	–	–	–	0.080±0.002	0.130±0.004	0.180±0.006	1.03±0.03	0.98±0.03	0.130±0.004	0.060±0.002	0.050±0.002	0.070±0.002	0.98±0.03
Anthocyanins (mg/L)													
**Myrtillin**	–	–	–	0.020±0.001	–	–	0.47±0.03	0.050±0.003	–	–	–	–	0.57±0.03
**Malvin**	–	–	–	0.010±0.001	–	–	0.040±0.004	–	–	–	–	–	0.060±0.006
**Cyanidin 3-*O*-(2"-xylosyl)glucoside**	–	–	–	–	–	–	0.040±0.006	0.020±0.003	–	–	–	–	0.040±0.006
**Chrysanthemin**	–	–	–	–	–	–	0.15±0.01	0.020±0.001	–	–	–	–	0.28±0.02
**Peonidin 3-*O*-glucoside**	–	–	–	0.09±0.01	–	–	0.61±0.07	0.06±0.01	–	–	–	–	0.69±0.08
**Oenin**	–	–	–	0.55±0.05	–	–	3.4±0.3	0.64±0.06	–	–	–	–	3.9±0.4
RSA (mmol TE/L)	0.39±0.03	0.44±0.01	0.42±0.02	1.97±0.03	3.17±0.01	2.68±0.04	16.09±0.02	18.71±0.02	1.86±0.01	1.64±0.02	1.26±0.03	0.82±0.01	12.03±
TPC (g GAE/L)	1.15±0.02	1.44±0.02	1.41±0.01	1.44±0.02	1.51±0.03	1.67±0.01	2.24±0.00	2.36±0.01	1.35±0.02	1.31±0.02	0.81±0.01	1.17±0.01	1.71±

Table S4. Positive ion MS^4 fragmentation data for the anthocyanins identified in wine samples.

Peak No	Anthocyanins	t_R, min	Parent ion, M^+ (m/z)	MS^2 Fragments, (% Base Peak)	MS^3 Fragments, (% Base Peak)	MS^4 Fragments, (% Base Peak)
1	**Delphinidin 3-*O*-glucoside (Myrtillin)**^{a}	4.50	465	304(15), **303**(100)	303(15), 285(10), 275(10), **257**(100), 247(15), 229(30)	239(5), 229(100), 213(25), 201(25), 173(20)
2	**Malvidin 3,5-di-*O*-glucoside (Malvin)**^{a}	4.62	655	494(15), **493**(100), 332(10), 331(70)	**331**(100)	316(80), 315(100), 299(90), 298(30), 287(70), 270(50)
3	**Cyanidin 3-*O*-(2"-xylosyl)glucoside**^{a}	4.67	581	288(10), **287**(100)	287(60), 269(25), 259(40), 241(50), 231(70), **213**(100)	195(10), 185(100), 167(10), 157(20), 141(20)
4	**Cyanidin 3-*O*-glucoside (Chrysanthemin)**^{a}	4.78	449	288(10), **287**(100)	287(60), 269(25), 259(40), 241(50), 231(70), **213**(100)	195(10), 185(100), 167(10), 157(20), 141(20)
5	**Delphinidin 3-*O*-glucoside-pyruvate**	4.84	533	372(10), **371**(100)	371(30), 353(10), 343(10), **325**(100), 315(10), 297(20)	297(100), 281(80)
6	**Petunidin 3-*O*-glucoside isomer 1**	4.88	479	318(10), **317**(100)	**302**(100), 274(5)	274(100), 246(10), 228(10), 218(10)
7	**Petunidin 3-*O*-glucoside-acetaldehyde**	5.25	503	342(10), **341**(100)	327(10), **326**(100), 309(10), 281(5)	309(50), 298(100), 282(20), 270(40), 253(20), 241(30)
8	**Peonidin 3-*O*-glucoside**^{a}	5.29	463	302(10), **301**(100)	287(10), **286**(100)	268(20), 258(100), 230(25), 202(5)
9	**Malvidin 3-*O*-glucoside (Oenin)**^{a}	5.35	493	332(10), **331**(100)	316(80), **315**(100), 299(90), 298(30), 287(70), 270(50)	313(90), 299(25), 287(100), 285(70), 257(60)
10	**Delphinidin 3-*O*-(6"-acetyl)glucoside**	5.47	507	304(10), **303**(100)	303(20), 285(10), **257**(100), 247(10), 229(30)	229(100), 213(25), 201(30), 173(15)
11	**Malvidin 3-*O*-glucoside-acetaldehyde**	5.62	517	356(15), **355**(100)	**339**(100), 322(40), 311(15), 294(45), 266(20), 202(10)	337(50), 321(35), 311(100), 309(95), 293(10), 281(20)
12	**Peonidin 3-*O*-glucoside-pyruvate**	5.67	531	370(15), **369**(100)	**354**(100), 326(10)	336(25), 326(100), 298(30), 253(30)
13	**Malvidin 3-*O*-glucoside-pyruvate**	5.76	561	400(10), **399**(100)	**383**(100), 367(70), 355(30), 338(80), 310(40), 264(15)	381(60), 365(40), 355(100), 337(15), 325(20)
14	**Petunidin 3-*O*-(6"-acetyl)glucoside**	5.78	521	318(10), **317**(100)	**302**(100), 285(30), 274(10)	285(10), 274(100), 260(5), 246(15), 218(5), 153(20)

15	**Malvidin 3-*O*-glucoside-acetone**	5.92	531	370(10), **369**(100), 325(5)	**353**(100), 336(20), 325(10), 308(25), 280(15)	351(60), 335(30), 325(100), 323(80), 295(25)
16	**Malvidin 3-*O*-(6"-acetyl)glucoside-acetaldehyde**	5.98	559	397(100), **355**(100)	**339**(100), 322(35), 311(15), 294(40), 266(20), 202(10)	337(40), 321(300), 311(100), 309(80), 281(20), 255(10)
17	**Malvidin 3-*O*-glucoside-8-ethyl-(epi)catechin**	6.00	809	647(5), 519(20), **357**(100), 341(5)	**341**(100), 327(60), 313(10), 296(10), 268(20), 205(10)	339(90), 313(80), 311(100), 283(50)
18	**Malvidin 3-*O*-(6"-acetyl)glucoside-pyruvate**	6.05	603	400(15), **399**(100)	**383**(100), 367(80), 355(40), 338(80), 310(40), 264(20)	381(60), 365(40), 355(100), 337(20), 325(20)
19	**Peonidin 3-*O*-(6"-acetyl)glucoside**	6.07	505	302(10), **301**(100)	287(10), **286**(100)	268(20), 258(100), 230(25), 202(5)
20	**Malvidin 3-*O*-(6"-acetyl)glucoside**	6.10	535	332(10), **331**(100)	316(80), **315**(100), 299(90), 298(30), 287(70), 270(50)	313(100), 299(30), 287(90), 285(80), 257(60)
21	**Delphinidin 3-*O*-(6"-*p*-coumaroyl)glucoside**	6.23	611	304(10), **303**(100)	303(20), 285(10), **257**(100), 247(10), 229(30)	229(100), 213(25), 201(30), 173(15)
22	**Petunidin 3-*O*-(6"-*p*-coumaroyl)glucoside-8-ethyl-(epi)catechin**	6.35	941	665(60), 633(5), **343**(100), 328(10)	**328**(100), 300(10)	300(100), 272(10), 254(5)
23	**Malvidin 3-*O*-glucoside-4-vinyl-(epi)catechin**	6.50	805	644(15), **643**(100)	**491**(100)	476(20), 463(100), 448(20), 430(70), 402(10)
24	**Malvidin 3-*O*-(6"-*p*-coumaroyl)glucoside-acetaldehyde**	6.63	663	356(15), **355**(100)	**339**(100), 322(40), 311(15), 294(45), 266(20), 202(10)	337(50), 321(35), 311(90), 309(10), 293(10), 281(20)
25	**Cyanidin 3-*O*-(6"-*p*-coumaroyl)glucoside**	6.67	595	288(10), **287**(100)	287(60), 269(25), 259(40), 241(50), 231(70), **213**(100)	195(10), 185(100), 167(10), 157(20), 141(20)
26	**Malvidin 3-*O*-(6"-*p*-coumaroyl)glucoside-8-ethyl-(epi)catechin**	6.69	955	803(5), 665(60), 647(5), **357**(100), 341(10)	**341**(100), 327(60), 313(10), 296(10), 268(20), 205(10)	339(90), 313(80), 311(100), 283(50)
27	**Delphinidin 3-*O*-glucuronide**	6.70	479	304(10), **303**(100)	285(50), 274(20), **257**(100), 247(30), 229(80), 165(60)	229(100), 201(10)
28	**Malvidin 3-*O*-(6"-*p*-coumaroyl)glucoside-pyruvate**	6.72	707	400(10), **399**(100)	**383**(100), 367(70), 355(30), 338(80), 310(40), 264(15)	381(60), 365(40), 355(100), 337(15), 325(20)
29	**Malvidin 3-*O*-glucoside-4-vinylcatechol**	6.77	625	464(15), **463**(100)	448(50), **447**(100), 430(15), 419(10), 402(20), 374(20)	447(50), 445(65), 429(20), 419(100), 417(95)

**30**	**Malvidin 3-*O*-(6"-*p*-coumaroyl)glucoside**	6.80	639	332(10), **331**(100)	316(80), **315**(100), 299(90), 298(30), 287(70), 270(50)	313(90), 299(25), 287(100), 285(70), 257(60)
**31**	**Peonidin 3-*O*-(6"-*p*-coumaroyl)glucoside**	6.83	609	302(10), **301**(100)	287(10), **286**(100)	268(20), 258(100), 230(25), 202(5)
**32**	**Peonidin 3-*O*-glucoside-4-vinylphenol**	6.92	579	418(20), **417**(100)	403(20), **402**(100), 374(10), 346(5)	385(20), 374(100), 357(15), 346(60), 329(30)
**33**	**Malvidin 3-*O*-glucoside-4-vinylphenol**	7.01	609	448(25), **447**(100)	432(50), **431**(100), 414(15), 403(10), 386(20), 358(20)	431(50), 429(65), 413(20), 403(100), 401(95)
**34**	**Petunidin 3-*O*-glucoside isomer 2**	7.10	479	318(10), **317**(100)	**302**(100), 285(30), 274(10)	285(10), 274(100), 260(5), 246(15), 218(5), 153(20)
**35**	**Malvidin 3-*O*-glucoside-4-vinylguaiacol**	7.12	639	448(10), **447**(100), 331(5)	**462**(100), 444(5), 433(5), 416(10), 388(10)	461(15), 447(80), 434(100), 429(35), 401(30), 373(25)
**36**	**Malvidin 3-*O*-glucoside-pyranone**	7.19	533	372(10), **371**(100)	356(15), **343**(100), 339(10), 311(30), 283(20), 255(5)	311(100), 283(60), 265(5), 255(15)
**37**	**Malvidin 3-*O*-(6"-acetyl)glucoside-4-vinylphenol**	7.28	651	448(25), **447**(100)	432(50), **431**(100), 414(15), 403(10), 386(20), 358(20)	431(50), 429(65), 413(20), 403(100), 401(95)
**38**	**Malvidin 3-*O*-(6"-*p*-coumaroyl)glucoside-4-vinylcatechol**	7.30	771	464(15), **463**(100)	448(50), **447**(100), 430(15), 419(10), 402(20), 374(20)	447(50), 445(65), 429(20), 419(100), 417(95)
**39**	**Malvidin-pyruvate**	7.42	399	382(20), **381**(100)	366(10), **353**(100), 337(10), 325(90), 297(60)	338(5), 325(100), 309(10), 297(25)
**40**	**Malvidin 3-*O*-(6"-*p*-coumaroyl)glucoside-4-vinylphenol**	7.55	755	448(25), **447**(100)	432(50), **431**(100), 414(15), 403(10), 386(20), 358(20)	431(50), 429(65), 413(20), 403(100), 401(95)
**41**	**Delphinidin**	8.60	303	303(5), 285(60), 275(20), **257**(100), 247(30), 229(80)	239(5), **229**(100), 201(10)	201(100), 187(15), 183(10), 173(15), 161(20), 145(15)
**42**	**Petunidin**	9.77	317	**302**(100), 287(20), 285(30)	285(20), **274**(100), 257(5), 246(10), 229(5), 153(30)	257(20), 264(80), 229(40), 218(10), 153(100)

[a] Confirmed using avaliable standard

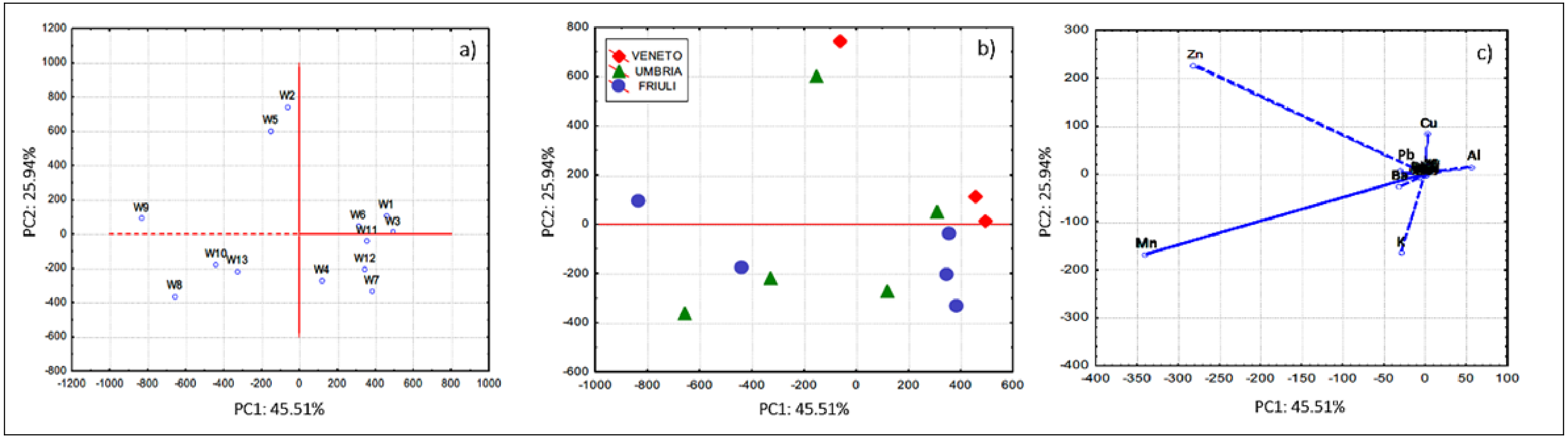

Figure S1. 1a): Scatter plot (PC1 vs PC2) obtained from PCA carried out on the element concentrations reported in Table 2; 1b) Scatter plot where the geographical origin is reported; 1c) Loading plot.

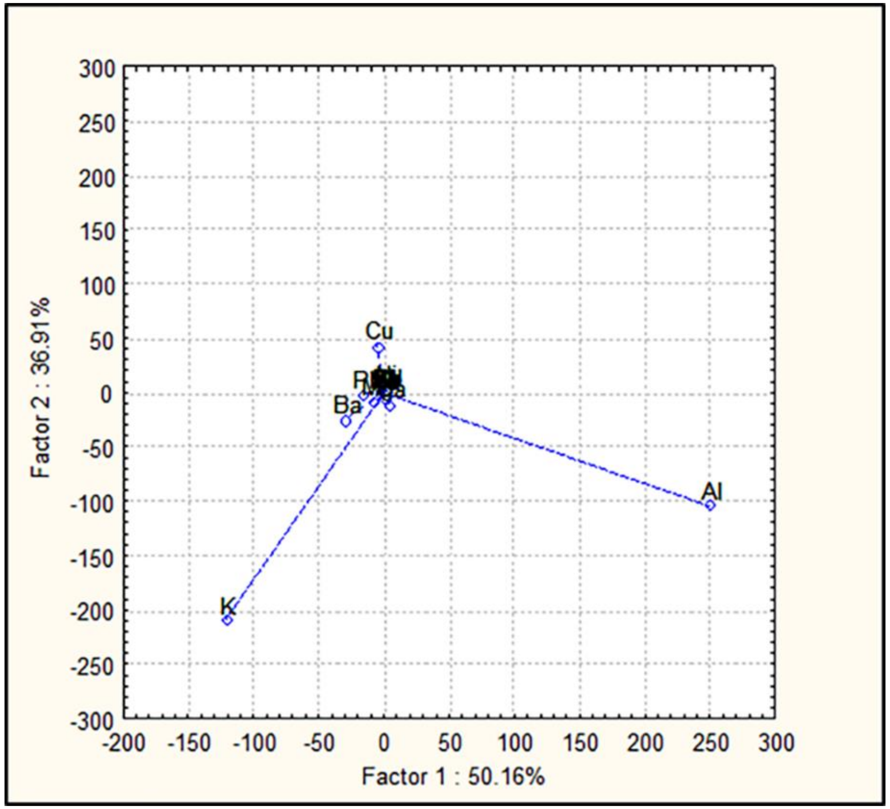

Figure S2. Loading plot obtained from PCA carried out on the original data set excluding from the calculation As, Zn and Mn.

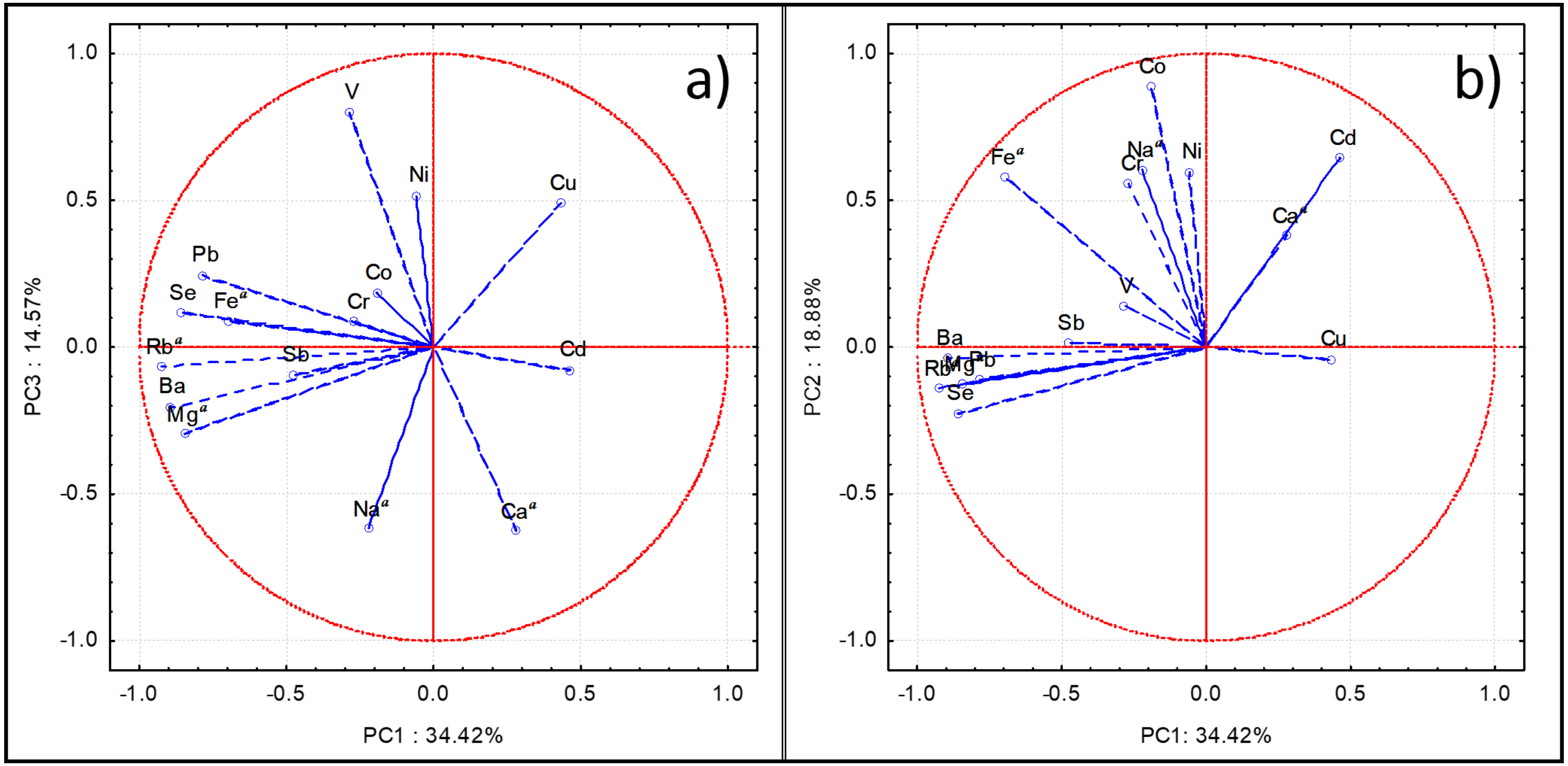

Figure S3. Loading plots obtained from PCA carried out on the original data set excluding from the calculation As, Zn, Mn, Al and K; a) PC1 vs PC23; b) PC1 vs PC2.

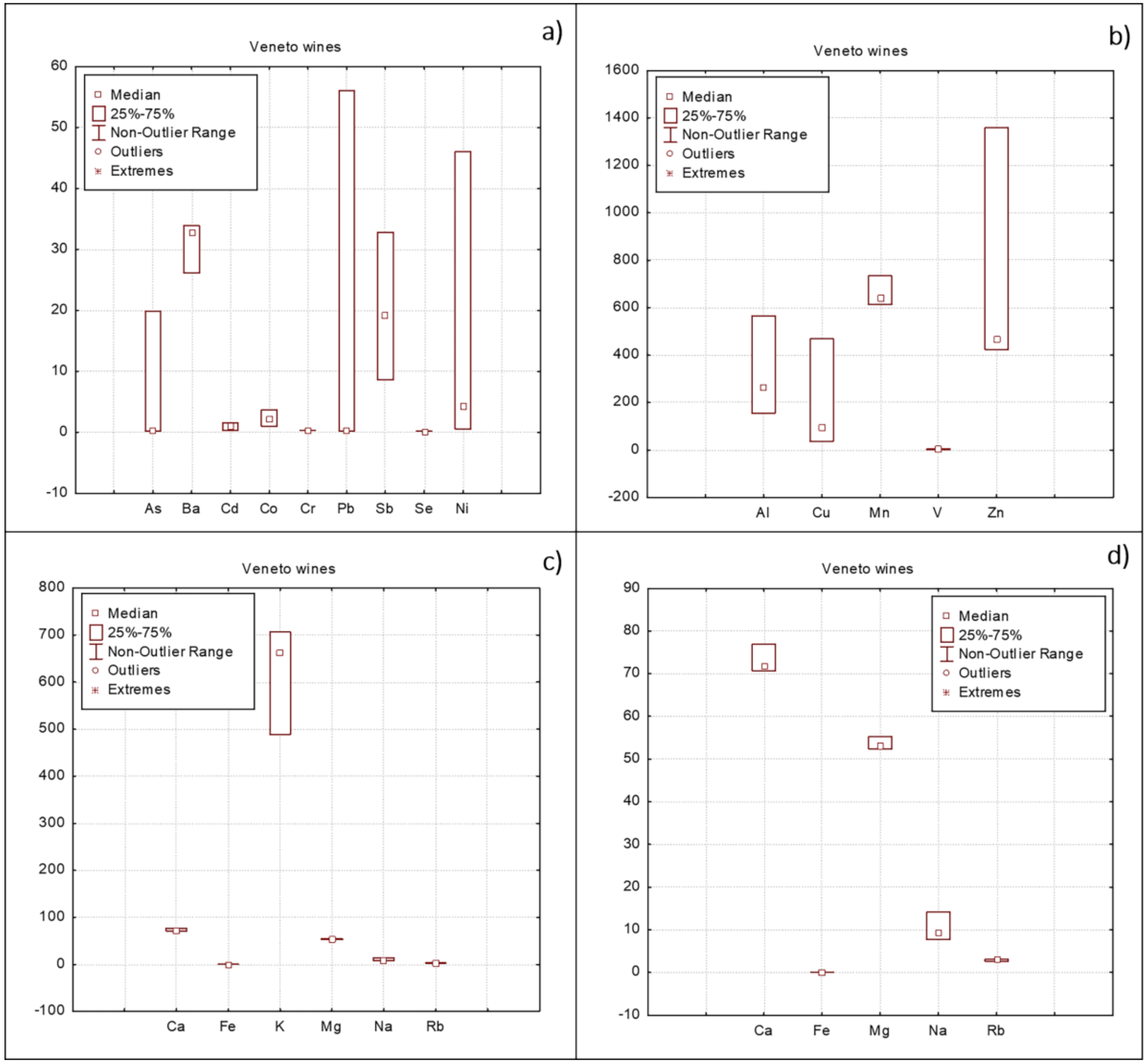

Figure S4. Box and whiskers plots for the elemental composition of Veneto wines; a), b), c) and d) graphs reports all the analyzed elements (in box d K has been eliminated to maximize the signals due to the other elements).

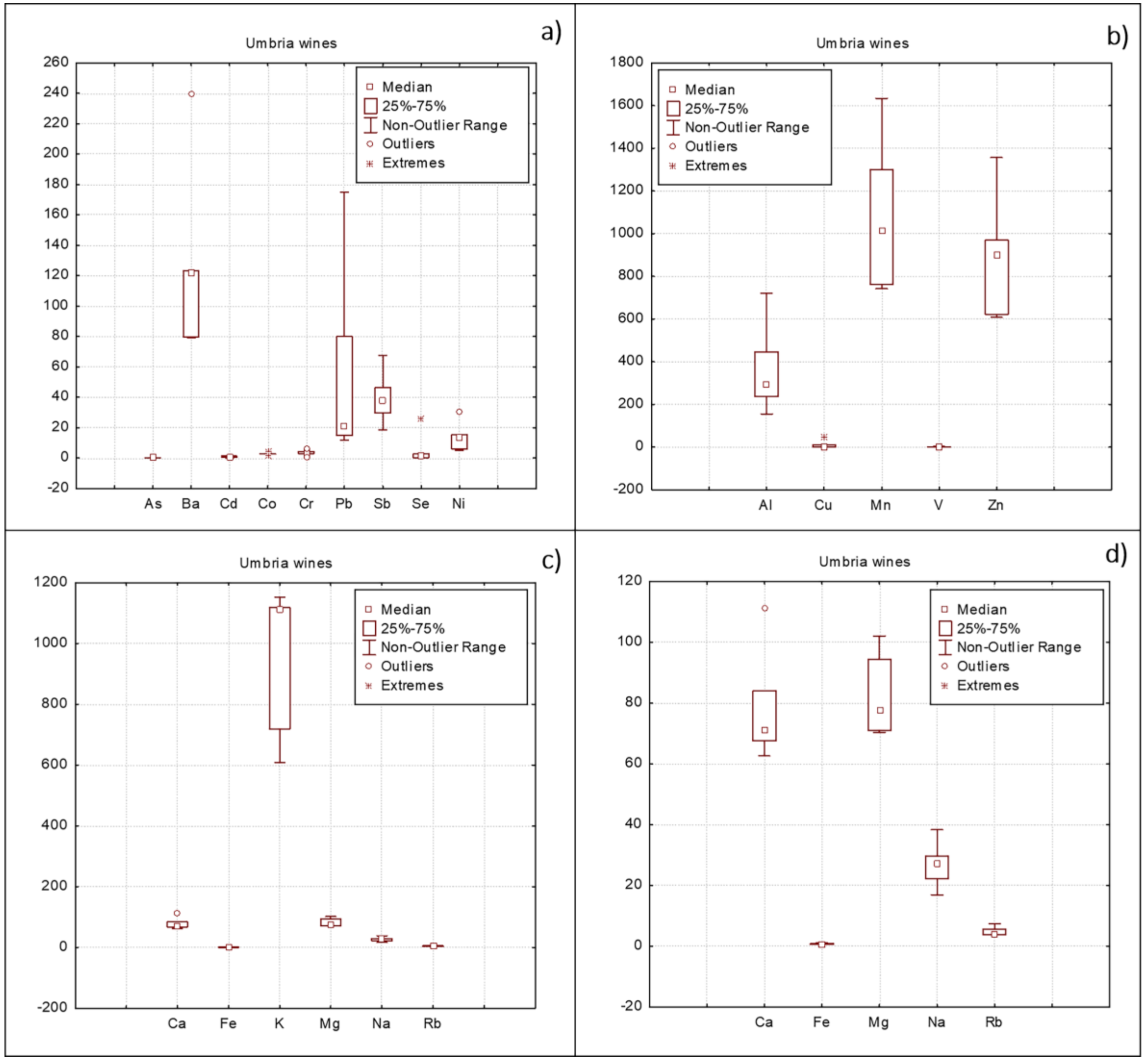

Figure S5. Box and whiskers plots for the elemental composition of Umbria wines; a), b), c) and d) graphs reports all the analyzed elements (in box d K has been eliminated to maximize the signals due to the other elements).

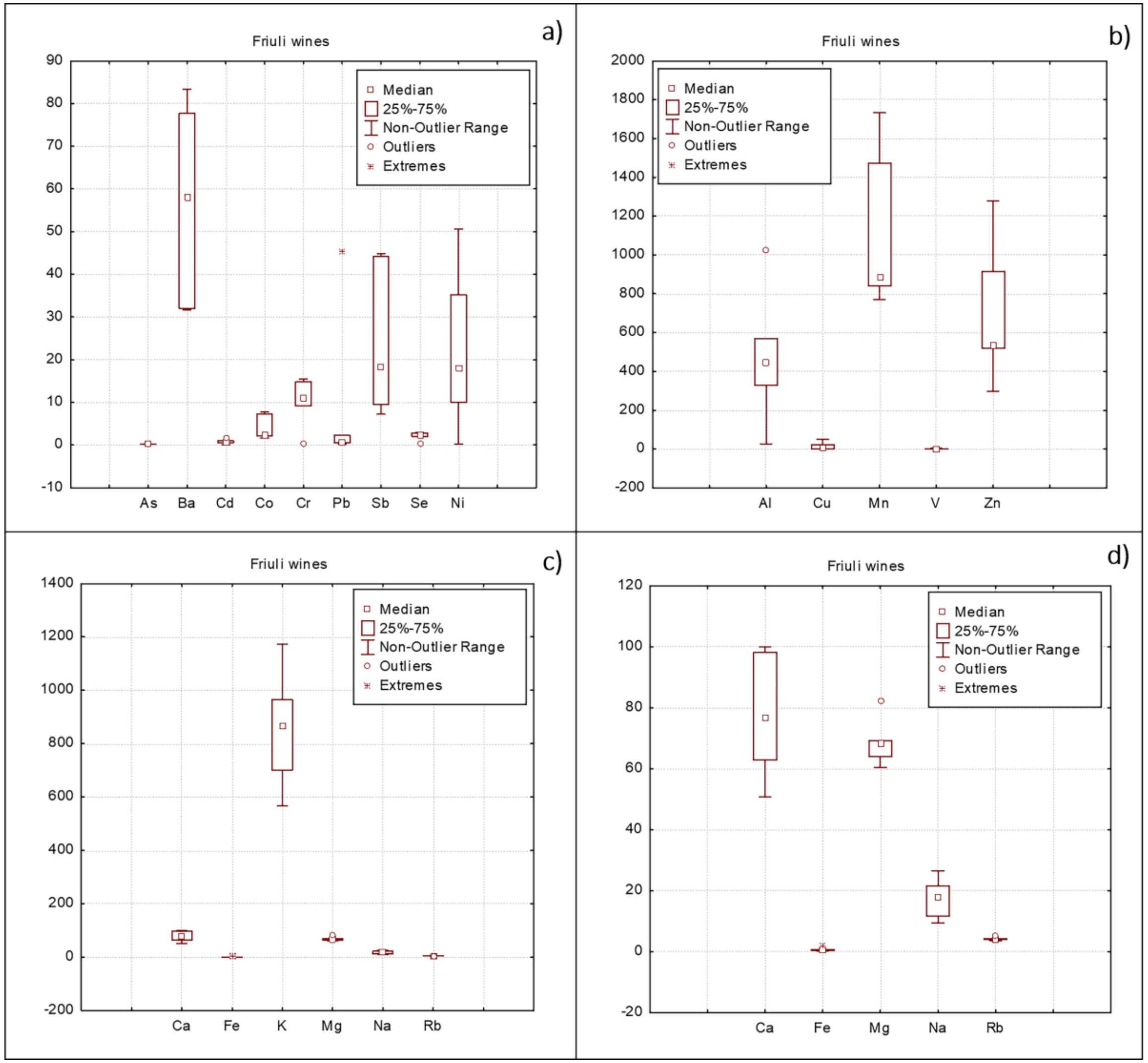

Figure S6. Box and whiskers plots for the elemental composition of Friuli wines; a), b), c) and d) graphs reports all the analyzed elements (in box d K has been eliminated to maximize the signals due to the other elements).
